# Constructing Curcumin-Based Biological Metal–Organic Frameworks (MOFs) for the Treatment of Alzheimer’s Disease Through the Pyroptosis Pathway

**DOI:** 10.3390/ijms27062871

**Published:** 2026-03-22

**Authors:** Fanshu Sun, Kangning Liu, Enpeng Xi, Yun Zhao, Nan Gao

**Affiliations:** Key Laboratory of Polyoxometalate and Reticular Material Chemistry of Ministry of Education, Faculty of Chemistry, Northeast Normal University, Changchun 130024, China

**Keywords:** Alzheimer’s disease, pyroptosis, curcumin, taxifolin, Zn–organic framework

## Abstract

Alzheimer’s disease (AD) is a chronic, progressive neurodegenerative disorder that presents as neuronal cell death caused by the pyroptosis pathway. Currently, curcumin is widely reported in the treatment of AD due to its dual inhibitory effects on NLRP3-associated inflammasome activation, but it suffers from poor bioavailability. Therefore, in this study, a highly stable curcumin-based Zn–organic framework (medi-MOF-1) loaded with taxifolin (TAX@medi-MOF-1) was presented to overcome the defect with a specific surface area of 2530.652 m^2^ g^−1^. The loaded TAX could further enhance the anti-inflammatory and antioxidant properties. In 5×FAD transgenic mice, TAX@medi-MOF-1 significantly improved cognitive and motor functions, reduced Aβ plaque deposition, and downregulated key pyroptosis proteins (NLRP3, caspase-1, and GSDMD-N). The dual-drug system exhibited synergistic effects, offering a promising multi-target therapeutic strategy for AD.

## 1. Introduction

Alzheimer’s disease (AD) is a chronic, progressive neurodegenerative disorder characterized by the deposition of β-amyloid protein (Aβ) and hyperphosphorylation of Tau protein [1,2,3]. Presently, AD is the leading cause of dementia, affecting over 50 million globally, with China having more than 13 million patients [4,5,6]. This progressive disorder destroys memory and cognitive functions, eventually impairing daily life [7,8,9]. Therefore, caregivers endure chronic stress, while medical costs and caregiving demand strain household budgets. Despite its prevalence, no cure exists. Current treatments focus on symptom management, and research highlights prevention through healthy lifestyles [10,11]. Addressing this crisis requires stronger support systems and global collaboration to lessen its impact on individuals and society.

Recent studies have shown that Aβ and Tau proteins cause neuronal cell death through the pyroptosis pathway, leading to the worsening of AD. In the early stage of pyroptosis, the NLRP3 (NOD-like receptor protein 3) will initially be activated by Aβ oligomers and Tau proteins [12], and the ASC (apoptosis-associated speck-like) protein is recruited to form inflammasomes [13,14,15,16]. Then, the inflammasomes cleave the pro-caspase-1 (pro-cysteinyl aspartate-specific proteinase-1) to obtain mature caspase-1, which affects two downstream pathways [17]. The first pathway involves cleaving Pre-interleukin-1β (IL-1β) to form mature IL-1β [18,19,20], and the other pathway involves cleaving gasdermin D protein (GSDMD) to form the p30 terminal protein (p30-GSDMD) [21,22,23,24]. p30-GSDMD is an oligomerization protein that can be located on the cell membrane, can open membrane pore channels, and can release various inflammatory factors, such as IL-1β, into the extracellular space and activate the pyroptosis pathway [25,26].

Currently, curcumin, resveratrol, and schisandrin are widely used in the treatment of AD due to their dual inhibitory effects on NLRP3-associated inflammasome activation [27,28,29,30]. However, due to their poor water solubility, the above-mentioned compounds are difficult to diffuse through cell membranes, resulting in low absorption efficiency and a reduction in the actual amount of drugs entering the bloodstream, greatly limiting their application [31,32,33]. In recent years, porous materials [34,35,36,37] have exhibited distinct advantages in drug delivery, attributable to their high specific surface area and tunable pore structure [38,39,40], which facilitate efficient drug loading and controlled release [9]. Curcumin serves as a representative compound that offers a viable chemical foundation for the development of metal–organic frameworks (MOFs) due to its potential for structural modification [41]. Additionally, its anti-inflammatory properties are linked to the underlying pathological mechanisms of AD. In our previous work, our group achieved material improvements by utilizing curcumin and Zn to synthesize a metal–organic framework (MOF) structure (named medi-MOF), with high specific surface area, good dispersibility, and good biocompatibility [42,43,44]. This also provides a material basis for the efficient use of curcumin to inhibit pyroptosis.

**Scheme 1:**
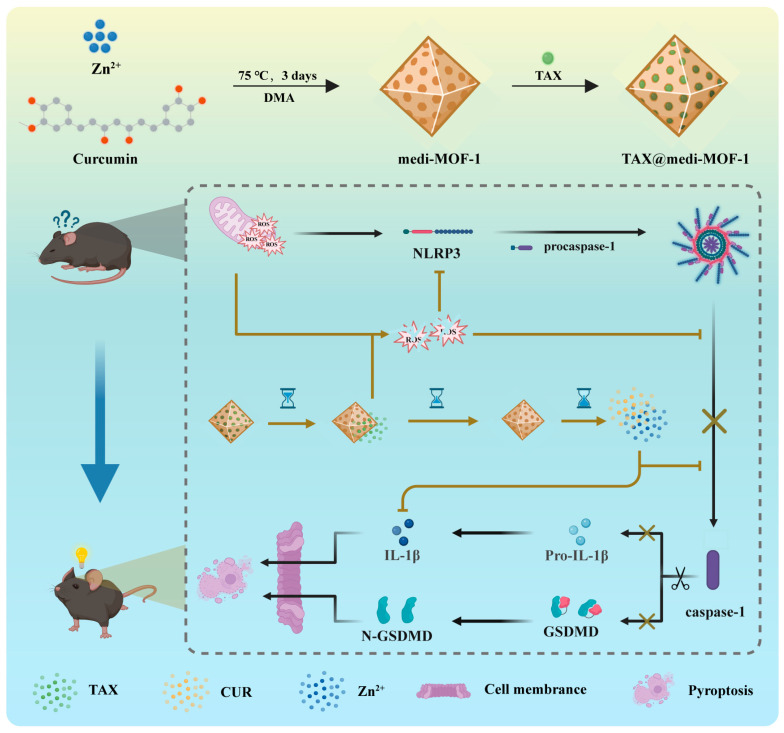
Schematic diagram of the synthesis process of TAX@medi-MOF-1; Schematic diagram of TAX@medi-MOF-1 in the treatment of Alzheimer’s disease by inhibiting the activation of NLRP3-related inflammasomes.

Furthermore, downstream inflammatory factors, IL-1β and reactive oxygen species (ROS), further amplify the effect of pyroptosis, exacerbating the death of nerve cells [45,46,47,48]. Therefore, another advantage of making curcumin into an MOF is that it can utilize its large specific surface area to further load anti-inflammatory and antioxidant drugs, thereby achieving multi-target inhibition of the apoptosis pathway. Herein, we employed Taxifolin (TAX) as a representative anti-inflammatory and antioxidant drug loaded in MOFs to create a dual-drug complex material (TAX@medi-MOF). The self-degradation of the MOF facilitates the release of curcumin and TAX, which enhances the intracranial environment in AD mice by modulating the NLRP3/caspase-1/GSDMD pyroptosis pathway, decreasing the levels of inflammatory factors and clearing ROS [49,50,51]. Notably, in transgenic mice, this TAX@medi-MOF system exhibits significant repair effects in spatial learning and memory (Scheme 1), and provides innovative insights for the treatment of AD through the pyroptosis pathway.

## 2. Results

### 2.1. Synthesis and Characterization of TAX@medi-MOF-1

Firstly, medi-MOF-1 was synthesized by hydrothermal synthesis technology. Curcumin and zinc acetate dihydrate (Zn(OAC)_2_·2H_2_O) were dissolved in a N, N-Dimethylacetamide-ethanol mixed solvent, stirred at room temperature for 4 h, and then heated in a closed container at 75 °C for 3 days. The synthesized medi-MOF-1 was washed successively with DMF, soaked in ultra-dry CH_2_Cl_2_ for 24 h, and then vacuum-dried at 100 °C for 8 h. The stability of the material directly affects the loading effect of the drug. Therefore, before conducting the drug loading and release experiments, we tested the stability of the material under different solvent conditions (Figure S1). Subsequently, TAX was loaded into medi-MOF-1 by solvent adsorption to obtain TAX@medi-MOF-1 composites. The absorbance of the ethanol solution of TAX at 289 nm was measured, the standard curve was plotted (Figure S2), and the loading rate of TAX was further calculated, resulting in a loading rate of 48.1% (Figure S3).

**Figure 1:**
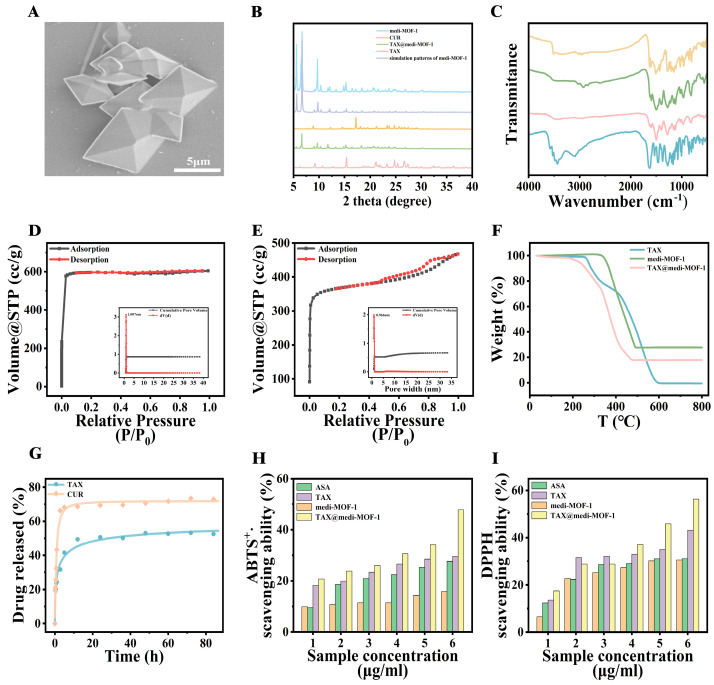
Structural characterization and antioxidant capacity of medi-MOF-1 and TAX@medi-MOF-1. (**A**) SEM image of TAX@medi-MOF-1; (**B**) XRD images of CUR, TAX, medi-MOF-1, TAX@medi-MOF-1 and the simulation patterns of medi-MOF-1; (**C**) FTIR images of TAX, medi-MOF-1 and TAX@medi-MOF-1 (Yellow: CUR; Green: medi-MOF-1; Pink: TAX@medi-MOF-1; Blue: TAX); (**D**) N_2_ adsorption–desorption isotherms of medi-MOF-1; (**E**) N_2_ adsorption–desorption isotherms of TAX@medi-MOF-1; (**F**) thermogravimetric analysis (TGA) of TAX, medi-MOF-1 and TAX@medi-MOF-1; (**G**) release process of CUR and TAX from TAX@medi-MOF-1; (**H**) determination of antioxidant capacity of different concentrations of TAX@medi-MOF-1 and corresponding contents of ASA, TAX, medi-MOF-1 by DPPH method; (**I**) determination of antioxidant capacity of different concentrations of TAX@medi-MOF-1 and corresponding contents of ASA, TAX, medi-MOF-1 by ABTS method.

Scanning electron microscopy (SEM) characterization indicated that the synthesized TAX@medi-MOF-1 presented an octahedral structure with a size of approximately 5 μm (Figure 1A). The X-ray diffraction (XRD) pattern shows that medi-MOF-1 has good crystallinity, proving the successful synthesis of medi-MOF-1 and the successful loading of TAX (Figure 1B). The synthetic crystals were analyzed by Fourier transform infrared spectroscopy (FTIR), and absorption peaks were observed at 3500–3200 cm^−1^, 1600–1450 cm^−1^, and 1300–1000 cm^−1^, corresponding respectively to O-H contraction vibration, benzene ring skeleton C=C stretching vibration, and C-O stretching vibration. The C-O stretching vibration absorption peak appears in the 1300–1000 cm^−1^ region. This is due to the vibration absorption of C-O bonds formed by multiple hydroxyl groups and other groups in the TAX molecule (Figure 1C).

The results of the N_2_ adsorption–desorption experiment of medi-MOF-1 showed that its Brunauer–Emmett–Teller (BET) specific surface area was 2531 m^2^ g^−1^, the total pore volume was 0.34 cc g^−1^, and the pore size was 1.007 nm, belonging to the Langmuir type I isotherm (Figure 1D). The BET specific surface area of TAX@medi-MOF-1 decreased to 1478 m^2^ g^−1^, and the pore size shrank to 0.966 nm, demonstrating the successful loading of the TAX molecule (Figure 1E). Subsequently, the thermal properties of the material were investigated using a thermogravimetric analyzer (TGA) in a nitrogen environment, with a temperature range of 30 to 800 °C. The medi-MOF-1 thermogravimetric curve confirmed that the porous skeleton has certain stability and maintains good stability when the temperature is below 350 °C. The mesothermal stage of 200–400 °C is the main thermal decomposition stage of TAX. Within this temperature range, the molecular structure of TAX begins to decompose, with chemical bonds breaking, generating some small-molecule compounds, such as carbon dioxide and water, leading to a rapid decline in mass. It can be seen that TAX has been successfully loaded into medi-MOF-1 (Figure 1F).

### 2.2. Antioxidant Testing of Drug Release and Antioxidant Systems

The drug-loaded samples were immersed in PBS at 37 °C (pH = 7.4), and the release amounts of TAX and curcumin in PBS were detected by liquid chromatography. The release curve indicates that TAX was released within 80 h, achieving a drug release rate of 70%. Meanwhile, medi-MOF-1 is unstable in PBS (Figure S2), and the degradation of the medi-MOF-1 host framework will be accompanied by the release of the drug. The degradation process of medi-MOF-1 occurred rapidly within the first four hours, and CUR in the material achieved a drug release rate of 70% (Figure 1G). Overall, the synergistic release of CUR and TAX indicates that the drug can be defined as a co-delivery system.

The antioxidant capacity of TAX@medi-MOF-1 was evaluated by two commonly used methods: 1, 1-diphenyl-2-trinitrophenylhydrazine (DPPH) and 2, 2-biazobis (3-ethylbenzothiazolin-6-sulfonic acid) diammonium salt (ABTS). The presence of curcumin in medi-MOF-1 endows the material with antioxidant properties. At the same concentration, the antioxidant capacity of TAX is higher than that of ascorbic acid. For TAX@medi-MOF-1, due to the presence of curcumin and TAX as two antioxidants, the antioxidant effect is the best (Figure 1H,I).

### 2.3. Biocompatibility and ROS Clearance of TAX@medi-MOF-1

**Figure 2:**
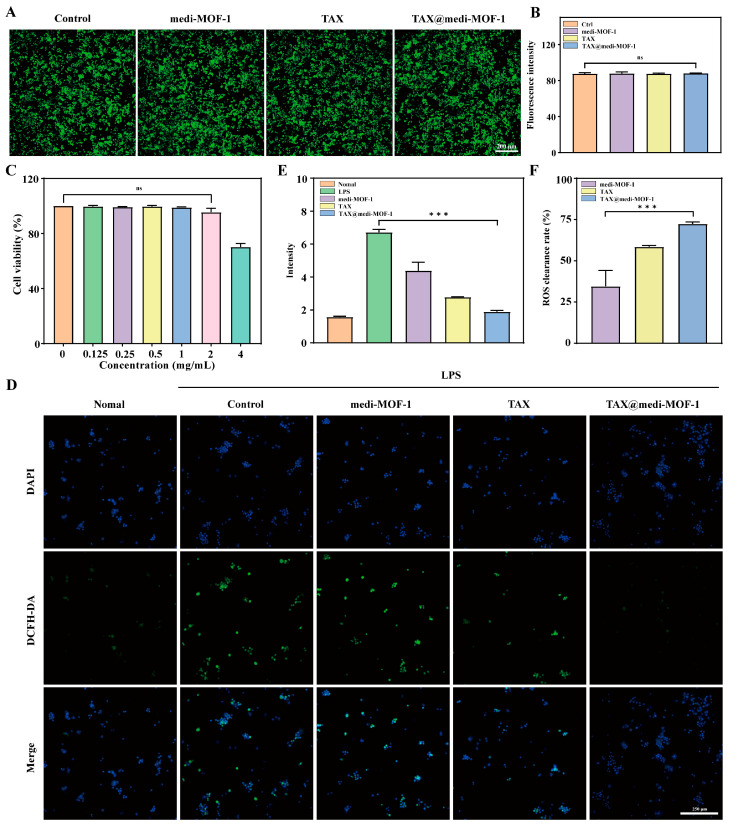
(**A**) Photographs of live/dead cell staining of RAW264.7 after different treatments; (**B**) summary of fluorescence intensity in (**A**); (**C**) cytotoxicity of TAX@medi-MOF-1 to RAW264.7; (**D**) medi-MOF-1, TAX, TAX@medi-MOF-1 representative image of ROS cleared from RAW264.7 cells (blue: DAPI, green: DCFH-DA); (**E**) summary of fluorescence intensity in (**E**); (**F**) medi-MOF-1, TAX, TAX@medi-MOF-1 ROS clearance efficiency. ns *p* > 0.05, and *** *p* < 0.001.

To assess the effects of medi-MOF-1, TAX, and TAX@medi-MOF-1 on cell viability, each material was co-cultured with RAW264.7 cells at a concentration of 2 mg mL^−1^ to evaluate cell viability and apoptosis status. Cytotoxicity was negligible across all groups (Figure 2A,B). The CCK8 assay was employed to examine the impact of TAX@medi-MOF-1 on cell proliferation. Following 24 h of exposure to TAX@medi-MOF-1 at concentrations ranging from 0 to 2 mg mL^−1^, RAW264.7 cell viability remained unaffected, indicating suitability for subsequent in vitro studies (Figure 2C). The intracellular reactive oxygen species (ROS) clearance effects of medi-MOF-1, TAX, and TAX@medi-MOF-1 were also investigated. Our findings demonstrated that 2 mg mL^−1^ of medi-MOF-1, TAX, and TAX@medi-MOF-1 effectively reduced excess intracellular ROS induced by LPS, with TAX@medi-MOF-1 exhibiting the highest clearance efficiency (Figure 2D–F). Both in vitro and in vivo experiments consistently indicate that TAX@medi-MOF-1 significantly reduces ROS levels in cells.

### 2.4. The Pyroptosis Pathway Improves the Motor Ability of AD Mice

We conducted long-term neurobehavioral tests on 5×FAD male mice to evaluate the sensory and motor abilities of the mice after material treatment. The entire zoological experiment period was one month (Figure 2A). Hemolysis experiments demonstrated that medi-MOF-1 and TAX@medi-MOF-1 do not rupture red blood cells (Figures S4 and S5). In the long-term behavioral test, the initial three weeks constituted the treatment phase. During this period, mice received intragastric administration every two days at a dosage of 20 mg kg^−1^, ensuring consistency of the drugs across the various treatment groups. The experimental results showed that during the administration period, there were no significant changes in the body weight and blood-related parameters of the mice (Figure 2B–D). For behavioral tests, we first used the rotating rod experiment to evaluate the motor coordination and balance abilities of mice. The results showed that the mice in the control group had better motor coordination and balance abilities, so they could stay on the spinning stick for a longer time. The time for mice in the AD group was shorter. After treatment, it was found that the rod time of mice in the AD + TAX group, AD + medi-MOF-1 group and AD + TAX@medi-MOF-1 group increased significantly, and there were significant differences within the groups (Figure S6). Subsequently, we utilized the open field experiment (OFT) to study the autonomous behavior of mice in novel environments and to explore the relationship between behavior and tension. By recording the activities of mice in this environment, such as the total distance moved, average moving speed, stay time, and the number of activities, the exploration behavior, anxiety level and adaptability of mice can be explored. The experimental results showed that the mice in the control group are more inclined to be active in the central area and are livelier. There were no significant differences in total movement distance, average speed and central time among the groups of mice (Figure 2E,F and Figures S7–S9), but they improved after treatment. TAX and TAX@medi-MOF-1 significantly increased the activity of AD mice.

**Figure 3:**
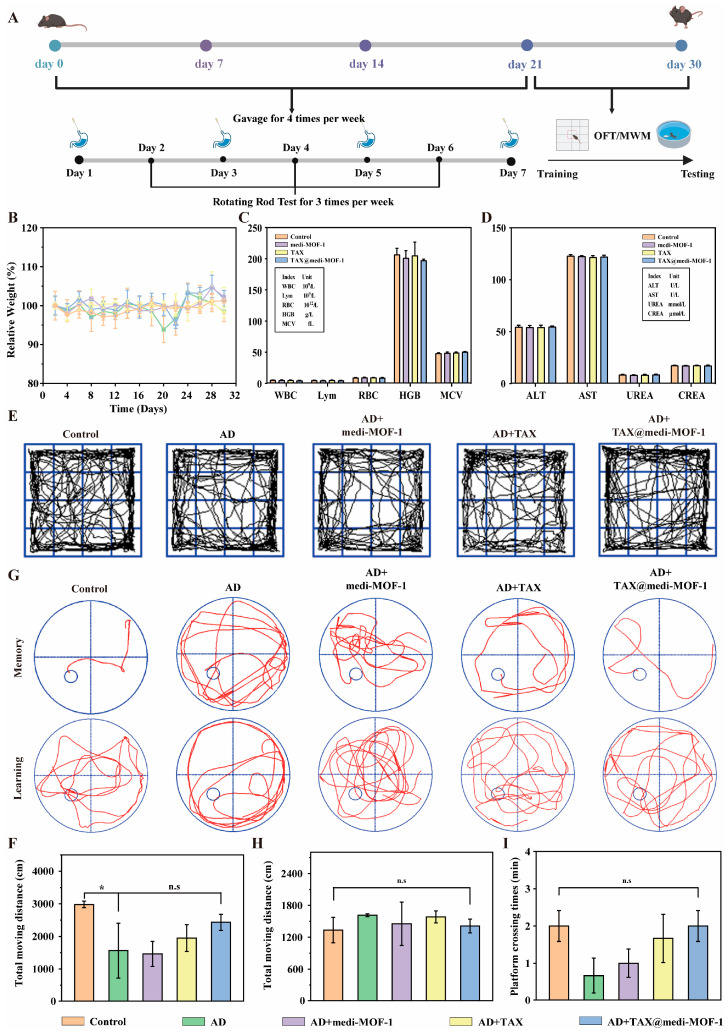
Behavioral tests of 5×FAD mice treated. (**A**) Timeline of the animal experiment; (**B**) images of weight changes during the behavioral tests; (**C**) blood routine indexes; (**D**) blood biochemistry indexes. Images of weight changes during the behavioral tests; (**E**) representative tracks of mice from five groups in the OFT; (**F**) total moving distance by 5×FAD mice in the OFT. The data are presented as mean ± SD, *n* = 6; (**G**) representative tracks of mice from five groups in MWM (blue circle: water maze edge, red: movement trajectory); (**H**) total moving distance by 5×FAD mice in the MWM. The data are presented as mean ± SD, *n* = 6; (**I**) time in platform quadrants by 5×FAD mice in the MWM. The data are presented as mean ± SD, *n* = 6. ns *p* > 0.05, * *p* < 0.05.

To assess the memory and cognitive functions of mice, we used the Morris Water Maze (MWM) to evaluate different treatment groups. After mice were diagnosed with AD, they showed impaired learning ability and the latency to reach the plateau was significantly prolonged (Figure S10). Compared with the AD group, treatment with medi-MOF-1, TAX and TAX@medi-MOF-1 improved the learning ability of mice and reduced the escape latency. Subsequently, the hidden platform was removed to evaluate the spatial memory ability of AD mice (Figure 3G). We evaluated the total movement distance, the time spent in the target quadrant, and the frequency with which mice in different experimental groups crossed the platform. Mice in the TAX group, TAX@medi-MOF-1 group, and medi-MOF-1 group exhibited shorter total movement distances compared to those in the AD group. This finding suggests an improvement in the cognitive abilities of the mice, allowing them to reach the target position more quickly (Figure 3H). Mice in the TAX@medi-MOF-1 group and the medi-MOF-1 group stayed in the target quadrant for a longer time than those in the AD group, indicating that medi-MOF-1 and TAX@medi-MOF-1 effectively restored spatial memory in mice (Figures S11 and S12). Meanwhile, the number of times the mice in the treatment group crossed the platform increased (Figure 3I). The combined results of the above behavioral tests indicated that TAX@medi-MOF-1 has a significant potential to reduce the cognitive level of AD mice.

### 2.5. Therapeutic Effect of TAX@medi-MOF-1 on 5×FAD Mice

**Figure 4:**
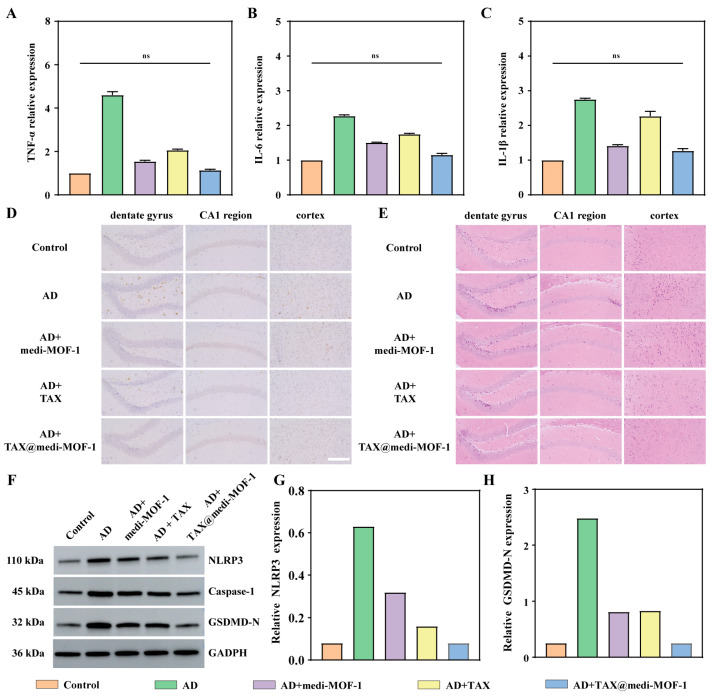
ELISA analysis of the secretion levels of (**A**) TNF-α, (**B**) IL-6, and (**C**) IL-1β in the brain of mice. The data are presented as mean ± SD, *n* = 3; (**D**) immunohistochemical staining of mice after treatment with different preparations, Bar = 200 μm; (**E**) H&E of mice after treatment with different preparations, Bar = 200 μm; (**F**) Western blot images; (**G**) quantitative Western blot analyses of NLRP3; (**H**) quantitative Western blot analyses of GSDMD-N. ns *p* > 0.05.

To further elucidate the impact of TAX@medi-MOF-1 on inflammatory mediators, we employed enzyme-linked immunosorbent assay (ELISA) to quantitatively assess key pro-inflammatory cytokines, specifically tumor necrosis factor-α (TNF-α), interleukin-1β (IL-1β), and IL-6. Following various treatments, the levels of these cytokines exhibited a reduction to differing extents. Notably, the TAX@medi-MOF-1 group demonstrated the most pronounced anti-inflammatory effect, characterized by the lowest concentrations of TNF-α, IL-1β, and IL-6. These results further underscore the anti-inflammatory properties of TAX@medi-MOF-1 (Figure 4A–C).

To elucidate the underlying regulatory mechanisms, we conducted immunohistochemical detection, H&E detection and Western blot detection on the mouse brain to observe the deposition of Aβ and the expression level of pyroptosis pathway-related proteins in the mouse brain. The immunohistochemical analysis results of Aβ in the brains of mice in different treatment groups showed that after combined treatment with TAX, medi-MOF-1 and TAX@medi-MOF-1, the number of Aβ plaques in the brains of mice in different treatment groups showed that after combined treatment with TAX, medi-MOF-1 and TAX@medi-MOF-1, the number of Aβ plaques in the brains of mice decreased to varying degrees. Compared with the AD + medi-MOF-1 group and the AD + TAX group, the TAX@medi-MOF-1 group significantly reduced the content of Aβ in the brains of 5×FAD mice, and the number of Aβ plaques was also significantly reduced (Figure 3D). The H&E results showed that after treatment in the medi-MOF-1 group, TAX group and TAX@medi-MOF-1 group, the tangles formed by the aggregation of hyperphosphorylated tau protein decreased, and the cell morphology returned to normal. Among them, the treatment effect was the best in the TAX@medi-MOF-1 group (Figure 3E). The Western blot test results showed that compared with the AD group, the levels of pyroptosis pathway proteins GSDMD-N, Caspase-1 and NLRP3 in the AD + TAX@medi-MOF-1 group were significantly lower, proving that the material treatment slowed down AD caused by the pyroptosis pathway to a certain extent (Figure 3F–H). Experimental results showed that long-term use of TAX@medi-1 can effectively reduce Aβ levels. In addition, histological analysis of the heart, liver, spleen and other organs of mice was conducted using hematoxylin–eosin (H&E) staining, and no obvious organ damage or inflammatory lesions were observed (Figure S13). This result further demonstrates the biological safety and good tolerability of TAX@medi-MOF-1.

## 3. Discussion

Alzheimer’s disease (AD) represents a significant global health challenge. Recent studies indicate that Aβ and Tau proteins can activate NLRP3 via the pyroptosis pathway, resulting in neuronal cell death and subsequent functional impairments. Concurrently, mitochondrial dysfunction and an imbalance in the antioxidant capacity of nerve cells may amplify pyroptosis signals [52,53], further exacerbating neuronal death and aggravating the progression of AD. Consequently, antioxidant therapy has emerged as the preferred approach in the clinical treatment of AD. The antioxidant system within organisms comprises antioxidant enzymes and antioxidants, which have become a focal point for simulating the antioxidant system in AD treatment. Recent years have seen a surge of interest in the application of metal–organic frameworks (MOFs) within the biomedical field. MOFs offer advantages such as well-defined structures, exceptionally high specific surface areas and porosity, adjustable pore sizes, and ease of chemical functionalization, positioning them as promising nanocarriers for drug delivery. Furthermore, certain MOF materials exhibit diverse enzymatic catalytic activities. By integrating MOF materials with antioxidant properties and antioxidant drugs, it is possible to construct an efficient and synergistic antioxidant system that effectively mitigates cognitive impairment in the brains of AD mice.

This study aims to develop an efficient antioxidant system. Drawing inspiration from the synthesis of metal–organic framework materials using drug molecules, we selected curcumin, an active drug molecule, as the ligand and combined it with biocompatible zinc ions to successfully synthesize the high-porosity drug-metal–organic framework compound medi-MOF-1. Consequently, we identified medi-MOF-1 as the drug carrier and incorporated taxifolin, a compound exhibiting both anti-inflammatory and antioxidant properties, into its pores to establish the antioxidant system.

Further investigations into the degradation process and drug loading capacity of medi-MOF-1 in vitro revealed that medi-MOF-1 functions not only as an effective drug carrier but also undergoes self-degradation, facilitating the release of curcumin from its framework. Curcumin demonstrates a significant inhibitory effect on the AD burn pathway. Experimental results indicate that the system can simultaneously release curcumin and taxifolin drug molecules, thereby achieving a synergistic drug effect. To assess the materials’ potential to enhance cognitive function, we employed a series of behavioral tests, including the rotating rod, open field, and water maze, utilizing the 5×FAD mouse model. The findings indicated that the antioxidant system exhibited remarkable antioxidant activity, effectively alleviating cognitive dysfunction and behavioral impairments in AD mice. Additionally, immunohistochemical analyses and measurements of related protein levels strongly corroborated the high efficiency of this antioxidant system in clearing Aβ plaques and its exceptional capacity to reduce the levels of charge-related protein factors.

In conclusion, we have developed an antioxidant system utilizing metal–organic frameworks as the core structural foundation. Comprehensive characterization and in-depth in vivo studies have demonstrated that these systems exhibit high antioxidant capacity and effective Aβ clearance. The antioxidant properties of Douglas fir inhibited the activation of NLRP3, while the anti-inflammatory effects of curcumin reduced levels of inflammatory factors, including IL-1β. This synergistic regulation significantly improved cognitive dysfunction in 5×FAD mice. The neuroprotective effect of TAX@medi-MOF-1 has been confirmed at this stage. In advanced AD, there is a marked loss of neurons accompanied by extensive neurodegenerative changes; some neurons retain functional plasticity. TAX@medi-MOF-1 may decelerate disease progression by inhibiting inflammatory responses and reducing oxidative stress, among other mechanisms, thereby mitigating damage to the remaining neurons. Despite the TAX@medi-MOF-1 system’s efficacy in drug loading and controlled release, its translational application is constrained by primary challenges. Future research should focus on optimizing this process via green synthesis technologies, which can lower energy consumption and costs while improving the biocompatibility and batch stability of MOFs, thereby establishing a foundation for their clinical application. This research not only offers a novel perspective for analyzing the pathological mechanisms of Alzheimer’s disease (AD) but also presents innovative strategies for constructing antioxidant systems that employ multi-mechanism synergy in the treatment of AD.

## 4. Materials and Methods

### 4.1. The Preparation Method of Materials

The following reagents were purchased from Merck Group Darmstadt, Germany. Curcumin (98%), Zn(OAC)_2_·2H_2_O (98%), N,N’-dimethylacetamide (99%), DMF (99%), CH_2_Cl_2_ (99%), TAX (98%), anhydrous ethanol. Curcumin (60 mg, 0.1629 mmol) and Zn(OAC)_2_·2H_2_O (20 mg, 0.0911 mmol) were added to the mixed solvent of N,N’-dimethylacetamide (4.0 mL) and anhydrous ethanol (1.0 mL). After stirring at room temperature for 4 h, it was heated in a sealed container at 75 °C for 3 days, and the product was red crystals. The crystals were collected, then washed with DMF (3 × 5 mL), and then the synthesized samples were soaked in ultra-dry CH_2_Cl_2_ for 24 h (three times), and the crystals were dried under vacuum at 100 °C for 8 h. TAX@medi-MOF-1 was prepared by solvent adsorption. Medi-MOF-1 and TAX were dispersed in anhydrous ethanol solution in a mass ratio of 1:2, stirred at room temperature for 12 h, and the red precipitate was collected by centrifugation and washed with ethanol three times. After vacuum drying, the final product was separated into red crystals.

### 4.2. Characterization of Materials

The scanning electron microscope (SEM) test was conducted using a JEOL-JSM-7600 instrument (JEOL, Tokyo, Japan) with an acceleration voltage of 5 kV. The powder X-ray diffraction test was conducted using the Dmax2200PC diffractometer (Rigaku, Tokyo, Japan), with a scanning range of 2–40° (2*θ*), Cu-Ka radiation, working parameters of 40 kV and 200 mA, and a scanning rate of 5° min^−1^. Fourier Transform Infrared spectroscopy (FTIR) analysis was performed using a Nicolet IS50 infrared spectrometer (Thermo Scientific, Waltham, MA, USA) and KBr particles, with a wavelength range of 4000–400 cm^−1^. The nitrogen adsorption–desorption isotherm was determined by a Quantachrome Autosorb-iQ2 gas adsorption instrument (Quantachrome, Boynton Beach, FL, USA) at 77 K and a relative pressure of 0–1 bar. Thermogravimetric analysis (TGA) was conducted using a METTLER-TOLEDO TGA/DSC3+ (METTLER TOLEDO, Zurich, Switzerland) thermogravimetric analyzer, with a temperature range of 30–800 °C, a heating rate of 10 °C min^−1^, and air atmosphere. The ultraviolet–visible absorption spectrum was determined by the VARIAN Cary-60 UV–visible spectrophotometer (Agilent Technologies, Santa Clara, CA, USA), with a wavelength range of 200–800 nm.

### 4.3. TAX@medi-MOF-1 Determination of Drug Loading Capacity and Encapsulation Efficiency

TAX was dissolved in ethanol. The standard curve of TAX was obtained by measuring the absorbance of ethanol solutions with concentrations of 0.01 mg mL^−1^, 0.008 mg mL^−1^, 0.006 mg mL^−1^, 0.004 mg mL^−1^, and 0.002 mg mL^−1^ at 289 nm, and the curve was plotted. medi-MOF-1 and TAX were dispersed in anhydrous ethanol solution at a mass ratio of 1:2, stirred at room temperature for 12 h, and centrifuged to collect the supernatant. The concentration of TAX in the supernatant was determined by ultraviolet spectrophotometry, and then the loading rate of TAX was calculated. The absorbance at 289 nm was detected by a UV-Vis spectrophotometer and substituted into the standard curve for calculation. Calculations were performed using the following formula:

Drug Loading Capacity (DLC)% = TAX mass in TAX@medi-MOF-1/medi-MOF-1 mass × 100%

Entrapment Efficiency EE% = mass of turmeric in TAX@medi-MOF-1/mass of curcumin input × 100%

Release of curcumin and TAX: At pH 7.4, 0.0177 g of vacuum-dried TAX@medi-MOF-1 was placed in 30 mL of a mixture of 0.5% Tween-20 and PBS, and shaken at 100 rpm at 37 °C. Then, 1 mL at the specified time point was drawn and replenished by an equal amount of fresh buffer. The aspirated liquid was centrifuged, and the supernatant was extracted. The concentrations of CUR and TAX in the supernatant were determined by liquid chromatography, and the release amounts of CUR and TAX were obtained.

### 4.4. Antioxidant Capacity Test of Dual-Drug System

The following reagents were purchased from Merck Group Darmstadt, Germany. DPPH· (≥98.5%), ABTS· (>99%). Determination of DPPH free radical scavenging activity: A 0.2 mM DPPH solution was prepared using ethanol. Subsequently, 1000 μL of the DPPH solution was combined with 1000 μL of various concentrations of medi-MOF-1, TAX@medi-MOF-1, TAX and ASA solutions. After thorough shaking and mixing, the solution was incubated at room temperature in the dark for 30 min. Ethanol served as the control group, and the absorbance was measured at 517 nm. The DPPH free radical scavenging activity was calculated as follows:

DPPH scavenging activity (%) = (A_control_ − A_sample_)/A_control_ × 100%

Determination of ABTS^+^ free radical scavenging activity: ABTS reagent with a concentration of 7 mM and potassium persulfate with a concentration of 2.45 mM were mixed in a volume ratio of 1:1, and incubated at room temperature in the dark for 12–16 h to prepare ABTS^+^ solution. Before use, the ABTS^+^ solution was diluted to achieve an absorbance value of 0.70 ± 0.02 at 734 nm. Then, 2000 μL of the diluted ABTS^+^ solution was added to 1000 μL of various concentrations of medi-MOF-1, TAX@medi-MOF-1, TAX and ASA solutions. The mixture was incubated at room temperature for 10 min, then the absorbance of the reaction system was measured at 734 nm. For the control group, the sample was replaced with ethanol. The ABTS^+^ free radical scavenging activity was calculated as follows:

ABTS^+^ scavenging activity (%) = (A_control_ − A_sample_)/A_control_ × 100%

### 4.5. Cell Culture

RAW264.7 cells were cultured in α-MEM medium containing 10% fetal bovine serum and 1% penicillin–streptomycin/fungizone at 37 °C and 5% CO_2_.

### 4.6. In Vitro Cytotoxicity Test

The following reagents were purchased from Wuhan Servicebio Biotechnology Co., Ltd., Wuhan, China. α-MEM medium, CCK-8 plus reagent, Calcein/PI cell viability and cytotoxicity assay Kit. RAW264.7 cell suspension (200 μL) was added to a 96-well plate to increase the cell count to 600 cells per well. After hatching, the cells were cultured at 37 °C for 24 h. The cells adhered, and the culture medium was discarded. Culture medium containing different concentrations of TAX@medi-MOF-1 (0, 0.125, 0.25, 0.5, 1, 2, and 4 mg/mL^−1^) was added to each well. After incubation at 37 °C for 24 h, 10 µL of CCK-8 plus reagent was added to each well. After incubation at 37 °C for 30 min, the absorbance of each well was measured at a wavelength of 450 nm using an enzyme-linked immunosorbent assay reader, and the cell survival rate was calculated:

Cell viability rate (%) = (OD_treatment_ − OD_control_)/(OD_blank_ − OD_control_) × 100%.

OD_treatment_: Absorbance of wells containing cells, CCK-8 solution, and TAX@medi-MOF-1 solution.

OD_blank_: Absorbance of wells containing culture medium and CCK-8 solution but no cells.

OD_control_: Absorbance of wells containing cells and CCK-8 solution but no TAX@medi-MOF-1 solution.

Live/dead cells were labeled using the Calcein/PI cell viability and cytotoxicity assay Kit, and observed and recorded using an inverted fluorescence microscope.

### 4.7. In Vitro ROS Clearance Experiment

The following reagents were purchased from Wuhan Servicebio Biotechnology Co., Ltd., Wuhan, China. α-MEM medium, LPS, PBS, DCFH-DA. RAW264.7 cells in the logarithmic growth phase were seeded at a density of 1 × 10^6^ cells per well into confocal glass-bottom culture dishes and incubated overnight in a temperature-controlled incubator at 5% CO_2_ and 37 °C. The cells were grouped as necessary, pretreated with LPS (20 ng mL^−1^), and subsequently co-incubated with 2 mg mL^−1^ TAX@medi-MOF-1 for 24 h. Following this, 1 mL DCFH-DA (10 μg mL^−1^) staining working solution was added and incubated at 37 °C in the dark for 30 min. After washing with PBS, 1 mL of Hoechst 33342 staining solution (110 μg mL^−1^) was added to each group and stained at room temperature for 10 min. Digital images were captured using an inverted fluorescence microscope.

### 4.8. Animal

In total, 5×FAD mice (male, 6–8 months) were purchased from JiangsuJicui Pharmachem Laboratory Animal Technology Co., Ltd., Nanjing, China. The rearing environment was maintained at a relatively constant temperature and humidity, with a 12 h light/dark cycle and unrestricted food and water supply. All research protocols involving animals were approved by the Animal Protection and Use Committee of Northeast Normal University. All experimental operations related to animals were in strict compliance with the “Environment and Facilities for Laboratory Animals” (GB14925-2010) [54], “Guidelines for Ethical Review of Laboratory Animal Welfare” (GB/T 35892-2018) [55], and the requirements of the Northeast Normal University Science and Technology Ethics Committee. All mice were acclimatized to the environment for 7 days before enrolling in the experiment. All animal experiments were supervised by the Animal Health and Use Committee of Northeast Normal University. Authorization Number: 202502098.

### 4.9. Behavioral Research on Mice

The control group consisted of six wild-type (WT) mice (Changchun Yisi Experimental Animal Technology Co., Ltd., Changchun, China). The experimental groups were the AD, AD + medi-MOF-1, AD + TAX, and AD + TAX@medi-MOF-1 groups. Twenty-four 5×FAD mice were stratified by body weight and randomly divided into 4 groups (6 mice in each group) to ensure that the average body weight of each group was balanced. During the experiment, all behavioral tests were conducted by independent laboratory personnel. Both the testers and the data analysts were double-blind to the animal group information to avoid subjective biases. Before treatment, baseline behavioral tests were conducted on all AD mice. The treatment protocol spanned 30 days and was divided into two stages. The final 9 days constituted the monitoring period, while the initial 21 days represented the treatment period. Prior to intragastric administration, the materials were ultrasonically crushed, and dispersions containing medi-MOF-1 (20 mg kg^−1^), TAX (20 mg kg^−1^), and TAX@medi-MOF-1 (20 mg kg^−1^) were administered bi-daily. Behavioral assessments were conducted every three days, with the open field test and the water maze test performed throughout the later stages of treatment, ensuring no overlap with other behavioral evaluations. Throughout the entire treatment duration, the weight changes in the 5×FAD mice were recorded daily. Blood and the main organs were collected for hematology.

### 4.10. Behavioral Science Test

Different behavioral tests were conducted on mice aged 6 to 8 months after treatment with various types of drug materials, including the stick test, water maze test and open field test. Before the test, all the animals were allowed to adapt to the test environment for 30 min each day for five consecutive days.

Rotating rod test: The ZH-600B (Anhui Zhenghua Biologic Apparatus Facilities Co., Ltd., Huaibei, China) rotating rod fatigue meter is a device that can accelerate from 5 rpm to 60 rpm within 360 s. The rod was initially rotated at a constant speed of 4 rpm. The mouse was placed on the rod, and an attempt was made to make the mouse walk forward on the rod to maintain balance. Once the mouse could walk forward at a speed of 5 rpm for a few seconds, the Start (Strat) button was pressed, and the rod accelerated from 5 rpm to 60 rpm within 360 s. Throughout the entire experiment, the rotator automatically recorded the time the mice spent on the rod and the time each mouse fell.

Morris Water Maze Test: Four training sessions each day for five days, with a 20 min interval between each session. The mice were randomly released from four compass positions, allowing them to swim and search for the platform for one minute. If the mouse failed to find the platform within one minute, it was placed manually on the platform and kept there for ten seconds. On the second day of learning, the mice conducted an exploration experiment in which the platform was removed. The mice were released from the northeastern starting point and allowed to swim freely for one minute. The paths taken by the mice were tracked and analyzed to determine the proportion of swimming time spent in the platform quadrant. Their behaviors were automatically recorded and then analyzed using the EthoVision video tracking system.

Open field test: Each mouse was placed in the center of a transparent plastic chamber (40 × 40 × 40.5 cm) and allowed to freely explore for 10 min. The test site was brightly lit. During each experiment, the behavior of the mice was automatically recorded and then analyzed using the EthoVision video tracking system.

### 4.11. Enzyme-Linked Immunosorbent Assay

The mouse TNF-α detection kit, mouse IL-1β detection kit and mouse IL-6 detection kit were purchased from Shanghai Enzyme linked Biotechnology Co., Ltd., Shanghai, China. After the behavioral test was completed, the mice were sacrificed to collect brain tissue. We assessed the levels of tumor necrosis factor-α (TNF-α), interleukin-1β (IL-1β), and interleukin-6 (IL-6) using enzyme-linked immunosorbent assay (ELISA). The specific experimental procedures were conducted in accordance with the manufacturer’s instructions, and the resulting data were recorded and analyzed.

### 4.12. Western Blotting

The following reagents were purchased from Wuhan Servicebio Biotechnology Co., Ltd., Wuhan, China. ACTIN (Rabbit), NLPR3 (Rabbit), GSDMD-N (Rabbit), Caspase-1 (Rabbit), fluorescent secondary antibody (goat), PVDF membrane (0.45 μm), 5×SDS-PAGE protein loading buffer, Rapid preparation of reagent kit, Protein-free rapid blocking solution, TBST buffer solution, PMSF, RIPA cracking solution. A portion of the brain tissue of mice was collected to obtain protein samples, which were lysed with phosphoprotein buffer and then centrifuged at 12,000 rpm for 10 min at 4 °C. Total proteins were isolated using SDS-PAGE and then transferred to a polyvinylidene fluoride (PVDF) membrane (Servicebio, G6047) at 300 mA for 30 min. The membrane was sealed with skimmed milk for 1 h and incubated overnight at 4 °C with NLRP3 (Servicebio, GB114320), GSDMD-N (Servicebio, GB114198-100) and Caspase-1 (Servicebio, GB11383-100) primary antibodies. After washing off the excess antibodies with TBST, the secondary antibody was added, and the samples were incubated at room temperature for 1 h. The excess antibodies were washed off again. Finally, chemiluminescence kits were used for detection, and the bands were visualized and analyzed using electrochemiluminescence (ECL) detection reagents (Servicebio, SCG-W3000).

### 4.13. Immunohistochemical Staining

The following reagents were purchased from Wuhan Servicebio Biotechnology Co., Ltd., Wuhan, China. 4% paraformaldehyde, Aβ antibody. A portion of the brain tissue of mice was fixed with 4% paraformaldehyde (Servicebio, G1101), then embedded in paraffin and sectioned. After dewaxing with xylene and gradient rehydration with ethanol, the tissues were evenly covered with 3% bovine serum albumin (BSA) and sealed at room temperature for 30 min. Aβ antibody (Servicebio, GB115755, 1:200) was added to the section and was incubated overnight at 4 °C. The sections were treated with AEC chromogenic substrates to observe immune complexes, and corresponding images were collected and analyzed using a 20× magnification microscope (Sunny, Yuyao, China, ICX41).

### 4.14. H&E Staining

The hearts, livers, spleens, lungs and kidneys of mice were paraffin-embedded and sectioned for histological analysis. The sections were stained with hematoxylin–eosin (H&E) and then observed under an optical microscope.

### 4.15. Statistical Analysis

All experimental data were processed using Origin 2024, GraphPad Prism 10.6 and ImageJ software (1.54p). All experimental data were expressed as mean ± standard deviation (SD). One-way analysis of variance was used to analyze the significance between groups. Significant differences in data were analyzed by * *p* < 0.05, ** *p* < 0.01, and *** *p* < 0.001.

## 5. Conclusions

In this study, we developed a novel dual-drug delivery system, TAX@medi-MOF-1, which targets the pyroptosis pathway and facilitates multi-mechanism synergistic treatment of AD by inhibiting NLRP3 inflammasome activation. This system employs curcumin, a candidate drug for AD treatment, as the core ligand to construct a metal–organic framework that demonstrates exceptional structural properties, including a BET specific surface area of 2531 m^2^ g^−1^, while maintaining structural stability upon efficient loading with 48.1% taxifolin. The dual-drug system achieved approximately 70% release rates for both CUR and TAX within 80 h, establishing an effective co-delivery platform. TAX@medi-MOF-1 exhibited superior antioxidant performance in both DPPH and ABTS assays, leveraging the combined effects of curcumin and taxifolin. In 5×FAD transgenic mice, TAX@medi-MOF-1 treatment significantly improved both motor coordination (rotating rod test) and cognitive functions (Morris Water Maze and open field tests). The composite material effectively reduced Aβ plaque deposition, decreased neurofibrillary tangles, and downregulated key pyroptosis pathway proteins (NLRP3, caspase-1, and GSDMD-N), demonstrating multi-target therapeutic efficacy. Histological analysis confirmed no significant organ damage or inflammatory lesions, indicating good biological tolerability. The TAX@medi-MOF-1 system represents a promising therapeutic approach that addresses multiple pathological mechanisms of AD, offering significant potential for clinical translation in treating this devastating neurodegenerative disorder.

## Data Availability

All data are contained within the article and Supporting Information.

## Supplementary Materials

The following supporting information can be downloaded at: https://www.mdpi.com/article/10.3390/ijms27062871/s1.

## Abbreviations

The following abbreviations are used in this manuscript:

MOFs	Metal–organic frameworks
AD	Alzheimer’s disease
NLRP3	NOD-like receptor protein 3
medi-MOF-1	Curcumin-based metal–organic framework
Aβ	β-amyloid protein
ASC	Apoptosis-associated speck-like
IL-1β	Interleukin-1β
GSDMD	Gasdermin D protein
p30-GSDMD	p30 terminal protein
ROS	Reactive oxygen species
TAX	Taxifolin
SEM	Scanning electron microscopy
XRD	X-ray diffraction
FTIR	Fourier transform infrared spectroscopy
BET	Brunauer–Emmett–Teller
TGA	Thermogravimetric analyzer
DPPH	1, 1-diphenyl-2-trinitrophenylhydrazine
ABTS	2, 2-biazobis (3-ethylbenzothiazolin-6-sulfonic acid) diammonium salt
OFT	Open field experiment
MWM	Morris Water Maze
DLC	Drug loading capacity
ECL	Electrochemiluminescence
BSA	Bovine serum albumin
H&E	Hematoxylin–eosin
PVDF	Polyvinylidene fluoride
