# Supplementary material for: Constructing Curcumin-Based Biological Metal–Organic Frameworks (MOFs) for the Treatment of Alzheimer’s Disease Through the Pyroptosis Pathway
Source: Int J Mol Sci. 2026 Mar 22;27(6):2871. doi: 10.3390/ijms27062871 (PMC13026492; doi:10.3390/ijms27062871)
Supplement: Supplementary file 1 [file ijms-27-02871-s001.zip › ijms-4191524-supplementary.pdf]

Supporting Information for

Constructing Curcumin-based Biological Metal-organic Frameworks (MOFs) for the Treatment of Alzheimer's Disease Through the Pyroptosis Pathway

Fanshu Sun [1†], Kangning Liu [1†], Enpeng Xi [1], Yun Zhao[1] and Nan Gao [1*]

[1] Key Laboratory of Polyoxometalate and Reticular Material Chemistry of Ministry of Education and Faculty of Chemistry, Northeast Normal University, Changchun 130024, China.

* Correspondence: gaon320@nenu.edu.cn (N.G.)

† These authors contributed equally to this work.

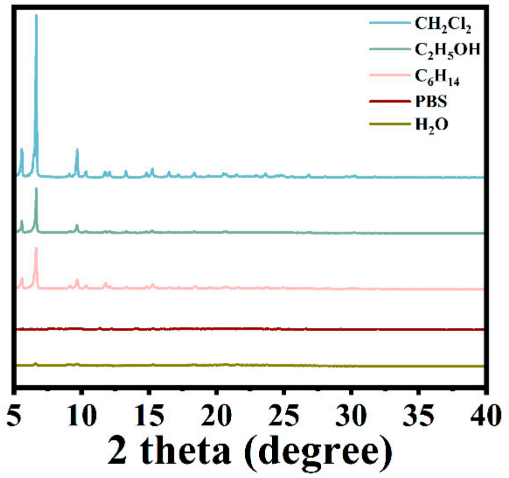

Supplemental Figure S1. XRD characterization of medi-MOF-1 under different solvent conditions.

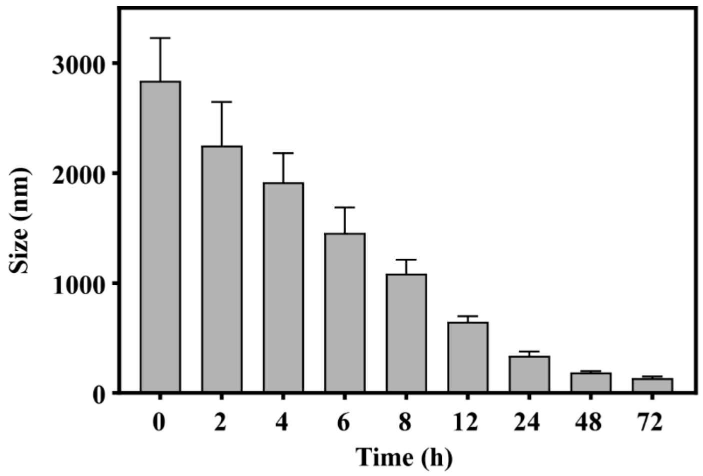

Supplemental Figure S2. The changes in particle size of medi-MOF-1 particles after being placed in PBS 37℃ for 3 days.

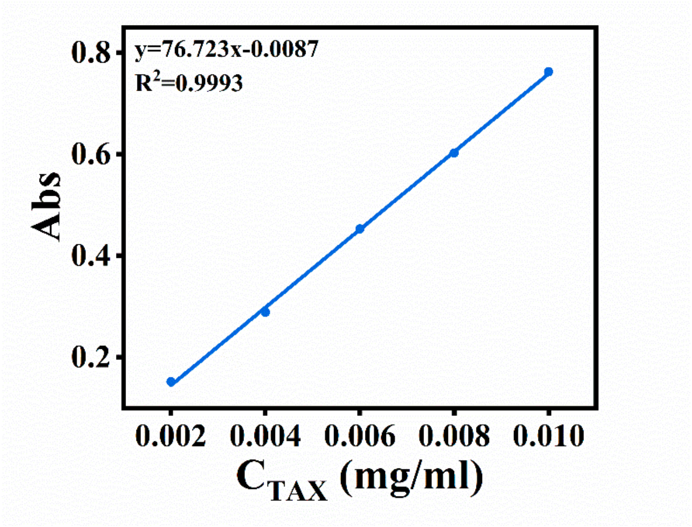

Supplemental Figure S3. Standard curve of TAX in ethanol solvent.

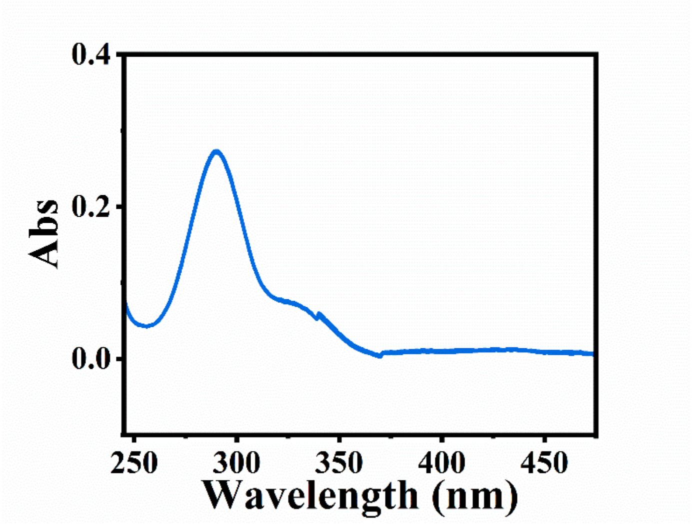

Supplemental Figure S4. The remaining TAX content in the supernatant after drug loading.

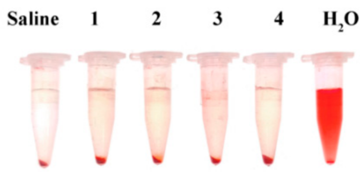

Supplemental Figure S5. Hemolysis test for the biosafety experiment of medi-MOF-1 (The unit of concentration is mg/mL).

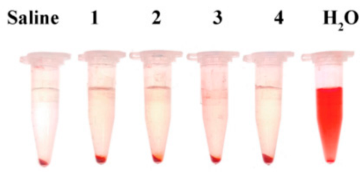

Supplemental Figure S6. Hemolysis test for the biosafety experiment of TAX@medi-MOF-1 (The unit of concentration is mg/mL).

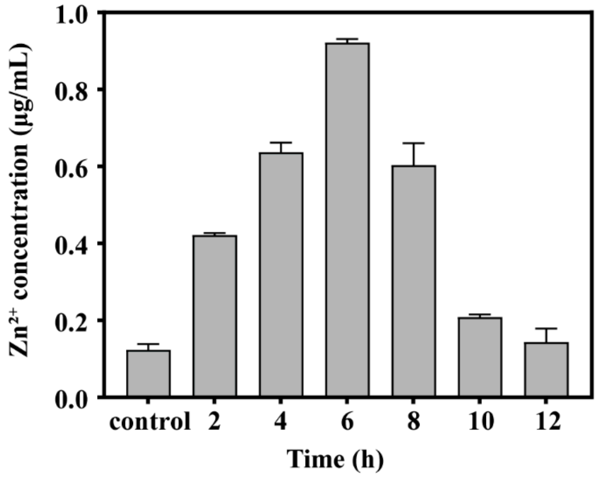

Supplemental Figure S7. The concentration of Zn^{2+} accumulated in the brain of mice over time.

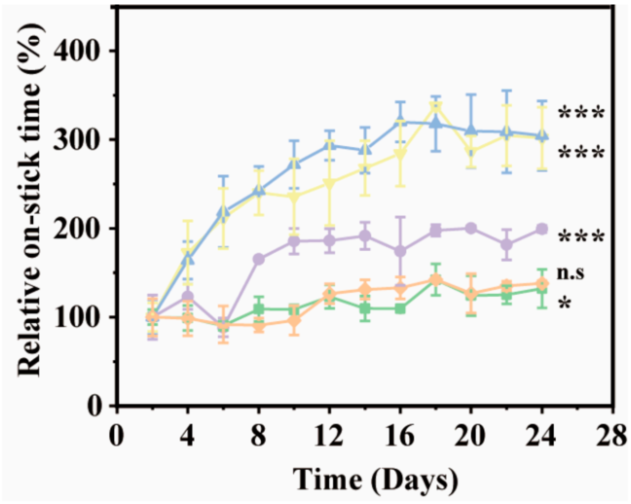

Supplemental Figure S8. Time of the mice on-stick. The data are presented as mean ± SD (n = 6).

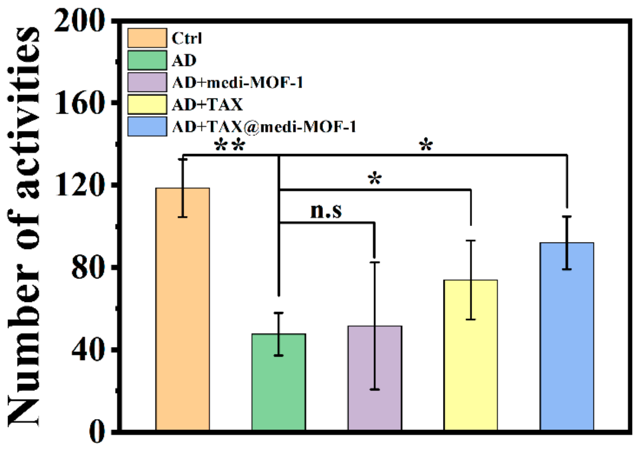

Supplemental Figure S9. Number of activities by 5×FAD mice in the OFT. The data are presented as mean ± SD (n = 6).

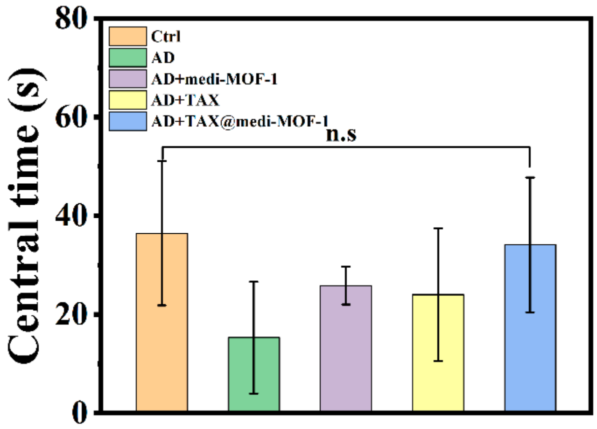

Supplemental Figure S10. Central time by 5×FAD mice in the OFT. The data are presented as mean ± SD (n = 6).

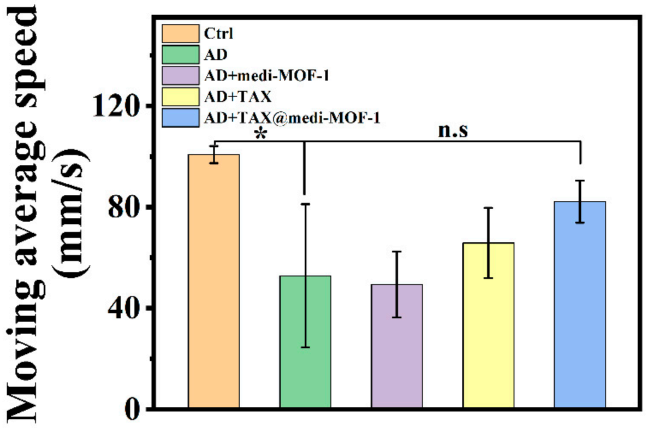

Supplemental Figure S11. Moving average speed by 5×FAD mice in the OFT. The data are presented as mean ± SD (n =6).

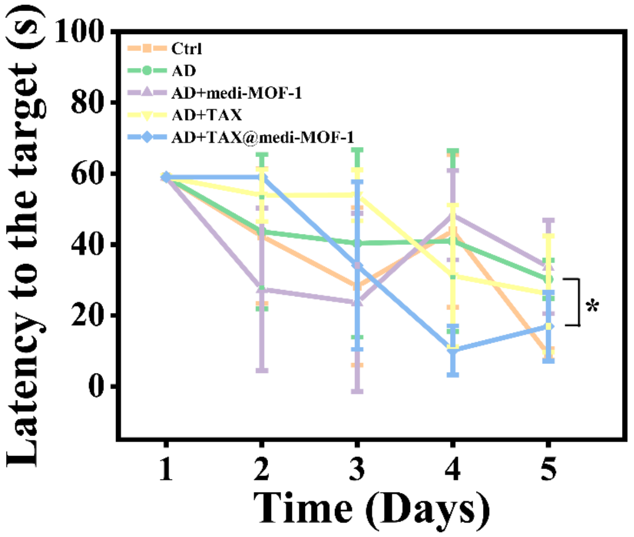

Supplemental Figure S12. Latency to the target by 5×FAD mice in the MWM. The data are presented as mean ± SD (n = 6).

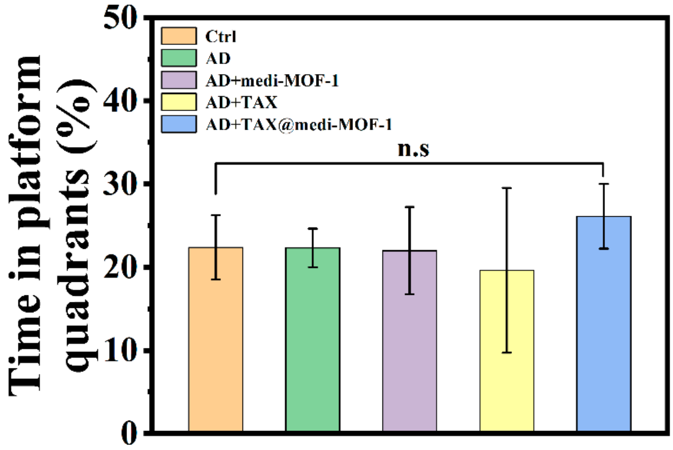

Supplemental Figure S13. Time in platform quadrants by 5×FAD mice in the MWM. The data are presented as mean ± SD (n = 6).

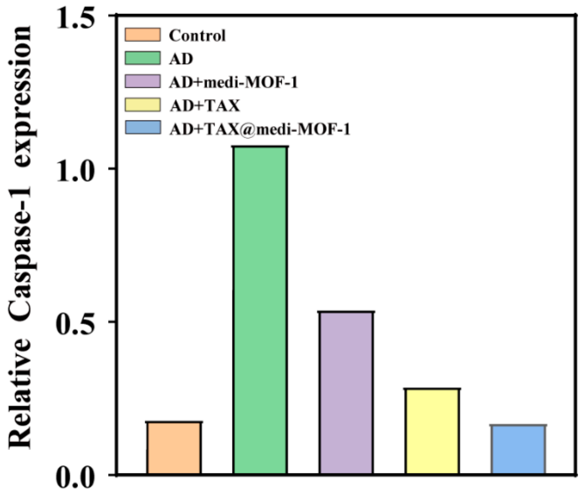

Supplemental Figure S14. Quantitative Western blot analyzes of Caspase-1.

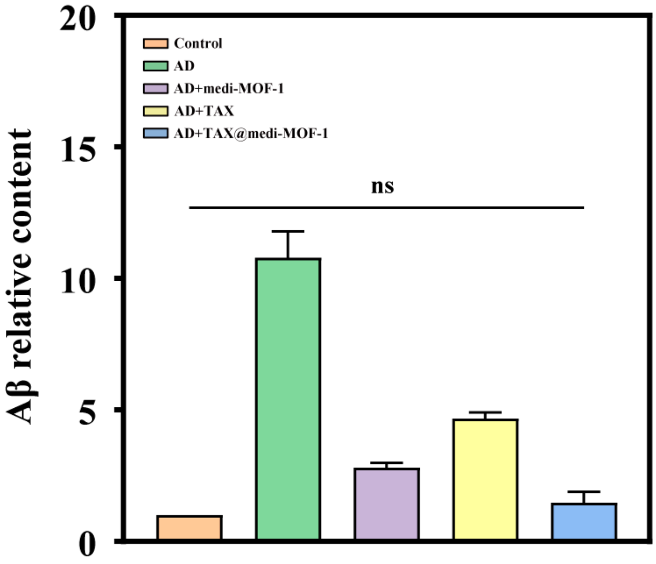

Supplemental Figure S15. The relative content of Aβ plaques in the dentate gyrus.

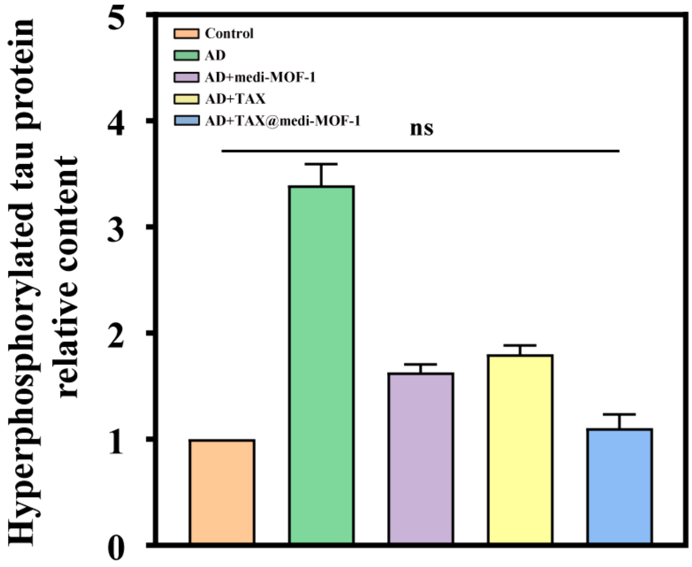

Supplemental Figure S16. The relative content of hyperphosphorylated tau protein in the dentate gyrus.

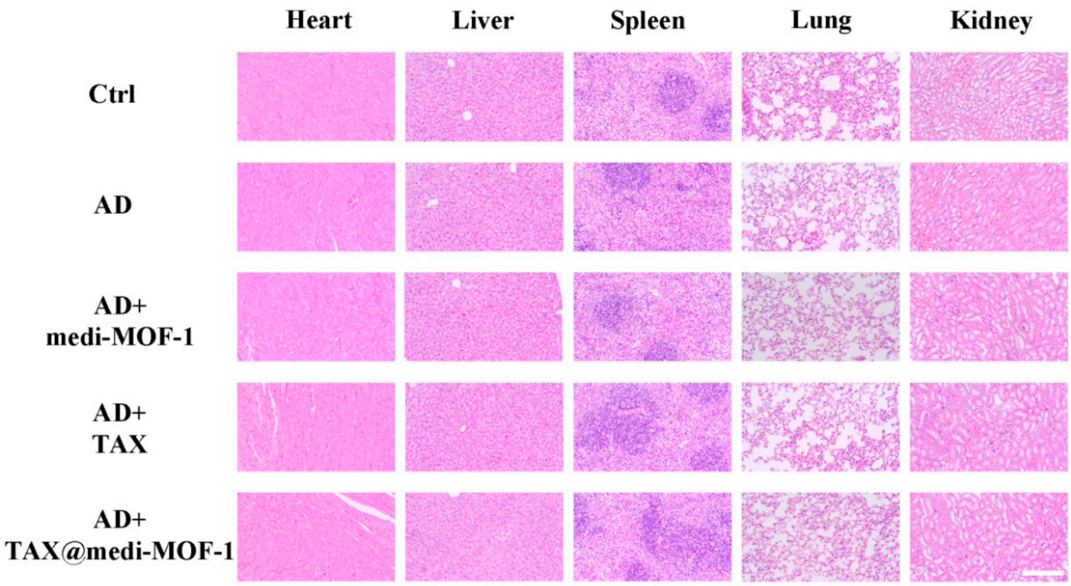

Supplemental Figure S17. H&E staining of major organs for biosafety experiments (scale bars are 200 μm).
